# Dental Caries Investigation in Children Controlled for an Educative and Preventive Oral Health Programme

**DOI:** 10.3290/j.ohpd.a44466

**Published:** 2020-07-04

**Authors:** Leila Maria Cesário Pereira Pinto, Eliane Mara Cesário Pereira Maluf, Luciana Tiemi Inagaki, Fernanda Miori Pascon, Regina Maria Puppin-Rontani, Elerson Gaetti Jardim Junior

**Affiliations:** a Professor, Pediatric Dentistry Specialties Center, Londrina State University, Londrina, Paraná, Brazil. Study design, data collection and all analyses.; b Professor, Department of Clinical Medicine, Federal University of Paraná, Curitiba, Paraná, Brazil. Statistical analysis.; c Professor, Department of Oral Medicine and Pediatric Dentistry, Londrina State University, Londrina, Paraná, Brazil. Interpretation of data, drafting of the manuscript and critical revisions.; d Professor, Department of Health Sciences and Pediatric Dentistry, Piracicaba Dental School, University of Campinas, Piracicaba, São Paulo, Brazil. Interpretation of data, drafting of the manuscript and critical revisions.; e Professor, Department of Health Sciences and Pediatric Dentistry, Piracicaba Dental School, University of Campinas, Piracicaba, São Paulo, Brazil. Interpretation of data, drafting of the manuscript and critical revisions.; f Professor, Pathology Department, São Paulo State University, Araçatuba, São Paulo, Brazil. Study design, data collection and all analyses.

**Keywords:** dental caries, child, oral health, preventive health services

## Abstract

**Purpose:**

To evaluate the association of dental caries with behavioural, socioeconomic and cultural factors; and *Streptococcus mutans* (SM) levels in the saliva and oral hygiene index of children aged 4 and 6 years old placed in an oral health programme.

**Materials and Methods:**

This study was an analytic cross-sectional oral health survey conducted over a 9-month period. A total of 466 children aged 4 and 6 years old were included for evaluation of SM levels in saliva, simplified oral hygiene index and dental caries activity.

**Results:**

High SM levels were associated with dmft index, toothbrushing without parental assistance, deficient oral hygiene and ingestion of sweet foods. Deficient oral hygiene was found in children aged 4 years old and with three or more siblings. Dental caries was associated with low family income, deficient oral hygiene, sucrose ingestion by children younger than three years old, bottle-feeding habit and low parental compliance.

**Conclusions:**

High SM levels in saliva, deficient oral hygiene and high frequency of sucrose ingestion had association with dental caries in children. Cultural, socioeconomic and behaviour factors indirectly influenced the onset of dental caries.

Dental caries is a complex disease caused by the presence of biofilm on teeth surfaces associated with high sucrose consumption,^26^ which in turn can contribute to the onset of cariogenic strains such as *Streptococcus mutans* (SM) and *Lactobacillus* sp, being also responsible for the biofilm’s acidogenic characteristics.^17^ In addition, if this biofilm is not removed efficiently from the dental surfaces, the cariogenic microbiological mass can mature, contributing to the onset of diseases.^16^ Others factors such as socioeconomic situation, educational level and health condition were considered mediating factors that could be indirectly associated with the onset and progression of dental caries.^26^ Thus, investments in preventive oral health programmes are necessary to control dental caries, especially in children.^4^

In relation to children, early childhood caries (ECC) is especially concerning for being defined as the presence of one or more carious lesions (cavitated or non-cavitated) in children younger than 72 months of age.^1^ ECC is considered a difficult disease to control because its onset is also associated with socioeconomic and cultural factors.^1^ Therefore, common habits such as the high frequency of ingestion of sweet foods could induce the child’s preference for sweets, thus contributing to the biofilm’s low pH and, consequently, increasing the risk of caries.^25,26^ In relation to the challenges to ECC control, oral health programmes that focus on an early approach, promoting dental care since the first year of life, seem to be a good proposal to make caregivers aware of the relevance of oral health, and this concept could be applied over the child’s life.^2,14^ Moreover, experiencing caries in primary dentition is a high risk factor for the development of new lesions,^7^ and it is also a predictor of the onset of the disease in permanent dentition.^15^

Many models of educative and preventive oral health programmes around the world have already been investigated,^3,4,11^ and the oral health programme of the Pediatric Dentistry Specialties Center of the Londrina State University (PDSC) (Londrina, Paraná, Brazil) has developed a pioneering work aimed at children. Founded in 1985, PDSC provides paediatric dentistry services from the first year of life until 72 months of age for the population of Londrina and nearby cities, focusing on educational and preventive approaches to oral health.^21,24^ Londrina is considered one of the most populated cities in the south of Brazil and PDSC is a centre of reference for the cities around it. Thus, investigating the factors associated with the onset of dental caries in a controlled sample could contribute to the promotion of knowledge about ECC.

For the reasons mentioned, the aims of this study were to verify the association of dental caries with: (a) behavioural, socioeconomic and cultural factors; and (b) SM levels in the saliva and oral hygiene index of children aged 4 and 6 years old, placed in one of PDSC’s Oral Health Programs. The hypotheses tested were: (a) behaviour, socioeconomic and cultural factors influence the onset of dental caries; (b) high SM levels and deficient oral hygiene are associated with the onset of dental caries.

## Materials and Methods

This study was an analytic cross-sectional oral health survey undertaken at PDSC with collaboration of the Pathology Department of the São Paulo State University (Araçatuba, São Paulo, Brazil). The study was approved by the Bioethics Committee of the Londrina State University and the São Paulo State University.

### Sample Selection

The sample’s definition was carried out based on PDSC appointments over a 9-month period, and 466 children aged 4 and 6 years old (medium of 47.2 months age with standard deviation of 2.6 and medium of 70.6 months of age with standard deviation of 2.0, respectively, were included. Of the total sample, 229 (49.1%) were female and 237 (50.9%) were male. Then, the children were distributed in two groups according to age: 212 children aged 4 years old (45.5%), and 254 children aged 6 years old (54.5%). These groups were selected because 4 years old represents the period of approximately 1 year after the primary second molars’ eruption; and 6 years old represents the period when the children leave the educational and preventive programme.

The children’s oral examination was conducted at PDSC during their regular appointments in the educative and preventive programme. An informed consent form was read and signed by the children’s parents/caregivers, and a questionnaire was used to collect behavioural, socioeconomic and cultural data. For this study, the exclusion criteria used were: children with disabilities, and children that had undergone antibiotic therapy in the last 3 months before the date of the saliva’s collection.^20^

### SM Evaluation in Saliva

Saliva samples were obtained after mastication of chewing gum using a microvacuum (Aspiramax MA 520, NS Industry, São Paulo, São Paulo, Brazil). The saliva sample was collected only one time for each child: in the morning (between 9:00 am and 11:00 am) or in the afternoon (between 3:00 pm and 5:00 pm), with an interval of at least 1 h after the last meal.^29^ Then, the saliva samples were put into eppendorfs and kept into an environment refrigerated with ice until processing to avoid the multiplication of microorganisms. The period between the time of collection and the procedure did not exceed 4 h. Then, 0.1 ml of saliva was diluted in 9 ml of prereduced anaerobically sterilised medium, and after this, the suspension was serially diluted in 10-fold steps.^27^ From this dilution, a 0.1 ml aliquot was inoculated in *Mitis salivarius* bacitracin sucrose agar (Becton, Dickinson and Company, Franklin Lakes, NJ, USA). The plates were incubated in an anaerobic environment (85% N_2_, 10% CO_2_, 5% H_2_) at 37°C for 72 h.^27^ Colony-forming unit (CFU) counting was conducted using a digital colonies counter (Comecta, Barcelona, Spain) and visual inspection. The saliva’s SM levels were categorised according to CFU numbers, in low (≥ 0 and < 10^4^), moderate (≥ 10^4^ and < 10^6^) and high (≥ 10^6^).^22^

### Simplified Oral Hygiene Index (OHI-S) and Dental Caries’ Evaluation

Clinical examinations were conducted by a single trained and calibrated examiner and a gold standard examiner as control (the intra- and interexaminer agreements’ Kappa index was 0.95 and 0.93, respectively). OHI-S was performed after saliva collection, using a hydroalcoholic basic fuchsine solution (Eviplac – Biodinâmica, Ibiporã, Paraná, Brazil) as a biofilm disclosing agent. Four teeth surfaces were examined: the vestibular surface of teeth 54, 61 and 82; and the lingual surface of tooth 75.^23^ A score range from 0 to 3 was used for the oral hygiene index,^10^ score 0 having represented no presence of biofilm on the surface; score 1 represented the presence of biofilm on no more than one-third of the surface; score 2 represented the presence of biofilm on more than two-thirds of the surface, and score 3 represented the worst situation, with more than two-thirds of the surface being covered by biofilm. After the oral hygiene index had been assessed, the teeth were cleaned with a toothbrush and dentifrice. Then, the dental caries’ visual evaluation was conducted by drying the teeth with an air spray under a dental light reflector. A mouth mirror and a dental explorer were used in situations of uncertain diagnosis. The dental caries was measured according to the dmft (decayed, missing and filled teeth) index,^30^ and the dental health conditions were categorised as: satisfactory (dmft = 1–4), deficient (dmft = 5–9) and very deficient (dmft ≥ 10).^18^

### Questionnaire

The questions applied evaluated the following variables: child’s age, number of siblings, birth order, parental education, parental professional occupation and family income. The oral health behaviour variables were: oral hygiene habits (up to what age the child received assistance during toothbrushing, time when the child started using dental floss, who is responsible for the child’s toothbrushing, child’s behaviour during toothbrushing in the evening), feeding habits, oral hygiene after bottle-feeding, age at which the child stopped being bottle-fed, daily frequency of ingestion of retentive and non-retentive foods with sucrose^9^; and factors associated with the onset of dental caries according to the perceptions of the children’s parents (questions about the parents’ perceptions in relation to preventive oral care, questions about whether they thought their child would develop dental caries one day, and questions about whether they thought it is important to take care of primary teeth). In addition, the parents’ difficulties following the recommendations of the oral educative and preventive programme were investigated, as were the difficulties to control the children’s sucrose ingestion before and after they had reached 3 years of age.

### Statistical Analysis

To estimate the risk of dental caries associated with the selected factors, the ODDS-RATIO (OR) was calculated with a 95% confidence interval (95% CI). Dental caries, salivary SM levels and OHI-S were the dependent variables; and the independent variables were the associations between the multiple factors. The multivariate analysis was conducted with logistic regression using software STATA 17 (Stata LP, College Station, TX, USA).

## Results

### Bivariate Analysis

Of the 466 children examined in this study, 354 were considered caries-free (81.6% with 4 years old and 71.3% with 72 months of age). The factors associated with dental caries with statistical significance (p < 0.05) were: child’s age, parents’ professional occupation, family income, age at the start of dental floss use, oral hygiene after bottle-feeding and sucrose frequency in the child’s diet. Of the 112 children that developed dental caries, 84% had satisfactory dental health condition (Table 1). Mothers with manual jobs had more children with caries (OR = 1.8; 95% CI:1.2–2.9) than those with intellectual occupations. Children who started flossing after 24 months of age had more dental caries (OR = 1.8; 95% CI:1.0–3.2) than those who started flossing before 24 months of age. Children who carried out oral hygiene after bottle-feeding had less dental caries than those who sometimes or never did it (OR = 1.8; 95% CI:1.0–3.2). As for family income, children in families with income lower than US$110.00 had more dental caries (OR = 2.1; 95% CI:1.2–3.6) than those whose family income was more than US$110.00. Therefore, when the chi-square tendency was applied to the family’s income, it showed prevalence of dental caries in children whose families had an income inferior to US$ 146.10. In relation to sucrose ingestion (Table 1), children who ingested retentive foods with sucrose more than 7 times/day had more dental caries (OR = 1.6; 95% CI:1.0–2.5) than those who ingested these foods 7 times/day or less.

**Table 1:** Distribution of children in relation to the presence and absence of dental caries using bivariate analysis with dental caries as dependent variable

Dependent variable: Dental caries						
Variables	With dental caries (n = 112)		Without dental caries (n = 354)		OR	95% CI
	n	%	n	%		
**Age** ^α^						
4 years old	39	18.4	173	81.6	1	
6 years old	73	28.7	181	71.3	1.8	1.1–2.9
**Mother’s professional occupation** ^α^						
intellectual job	58	19.8	235	80.2	1	
Manual handling job	54	31.2	119	68.8	1.8	1.2–2.9
**Family income (in US$)** ^α^						
≥ 110.00	80	21.2	297	78.8	1	
< 110.00	32	36.0	57	64.0	2.1	1.2–3.6
**Age at the start of dental floss use** ^α^						
< 24 months old	20	16.7	100	83.3	1	
≥ 24 months old	92	26.6	254	73.4	1.8	1.0–3.2
**Oral hygiene after bottle-feeding** ^*^ ^α^						
Yes	68	20.2	268	79.8	1	
no, sometimes	27	31.8	58	68.2	1.8	1.0–3.2
**Age at which bottle-feeding habit was abandoned**						
≤ 4 years old	91	22.6	311	77.4	1	
> 4 years old	21	32.8	43	67.2	1.7	0.9–3.1
**Daily frequency of ingestion of retentive foods with sucrose** ^α^						
≤ 7 times/day	60	20.8	228	79.2	1	
> 7 times/day	52	29.2	126	70.8	1.6	1.0–2.5
**Questionnaire answered by the parents:**						
**Do you think your child will have dental caries someday?** ^α^						
No	43	18.3	192	81.7	1	
Yes	69	29.9	162	70.1	1.9	1.2–3.0
**Is primary dentition care necessary?** ^α^						
Yes	104	23.2	345	76.8	1	
No	8	47.1	9	52.9	3.0	1.0–8.7
**Did you have difficulties following the recommendations?** ^α^						
No	81	21.7	292	78.3	1	
Yes	31	33.3	62	66.7	1.8	1.1–3.1
**Did you follow the recommendations on sucrose intake for children < 3 years of age?** ^**^^α^						
Yes	66	21.4	243	78.6	1	
No/Sometimes	23	47.9	25	52.1	3.4	1.7–6.7
**Did you follow the recommendations on sucrose intake for children ≥ 3 years of age?** ^***^^α^						
Yes	50	19.2	211	80.8	1	
No/Sometimes	29	44.6	36	55.4	3.4	1.8–6.4

Symbol (^α^) means statistically significant association (p < 0.05).

* total that answered about oral hygiene after bottle-feeding: 421.

** total that followed the recommendations on sucrose intake for children under 3 years of age: 357.

*** total that followed the recommendations on sucrose intake for children older than 3 years of age: 326.

Regarding the questionnaire given to the parents/guardians (Table 1), the caregivers who answered that their children could have dental caries someday had children with more dental caries (OR = 1.9; 95% CI:1.2–3.0) than those who believed their children would never develop the disease. Dental caries was also more prevalent in children whose parents did not believe that primary dentition care was necessary (OR = 3.0; 95% CI:1.0–8.7) and had difficulties following PDSC’s recommendations (OR = 1.8; 95% CI:1.1–3.1). It is important to note that parents who regularly followed the recommendations on sucrose intake had a high percentage of children free of dental caries (children younger than 3 years of age: 78.6%; children older than 3 years of age: 80.8%).

In relation to the salivary SM levels, of the total sample studied, 1.9% showed low levels, 63.3% moderate levels and 34.8% high levels of prevalence. High SM levels had statistically positive association with very bad/deficient oral hygiene (OR = 2.1; 95% CI:1.3–3.2) (Table 2), toothbrushing performed by the child and parents (OR = 1.9; 95% CI:1.2–3.0), ingestion of retentive sweet foods more than 7 times a day (OR = 1.6; 95% CI:1.1–2.4) and dmft index higher or equal to 5 (OR = 1.9; 95% CI:0.7–5.5). When oral hygiene quality was analysed (Table 3), the children aged 4 years old had a more deficient oral hygiene (OR = 2.1; 95% CI:1.4–3.3) than those aged 6 years old; and a tendency of low SM levels was found in children with regular (OR = 1.3; 95% CI:0.7–2.4) oral hygiene (Table 4). When the family’s oral healthcare was analysed, children with more than three siblings (OR = 1.9; 95% CI:1.1–3.4) and children who received oral hygiene support up to 4 years old. (OR = 1.5; 95% CI:1.0–2.4) had deficient oral hygiene (Table 3). Table 4 shows the high tendency of dental caries in children without parental help during toothbrushing.

**Table 2:** Distribution of children using bivariate analysis with *Streptococcus mutans* level in saliva (CFU/mL) as dependent variable

Dependent variable: *Streptococcus mutans* level in saliva						
Variables	CFU ≥ 10^6^/mL (n = 162)		CFU < 10^6^/mL (n = 304)			
	n	%	n	%	OR	95% CI
**Oral hygiene** ^α^						
Regular, satisfactory	107	32.6	243	69.4	1	
Deficient, very deficient	55	47.4	61	52.6	2.1	1.3–3.2
**Who performs the toothbrushing?** ^*^^α^						
Parents	36	25.4	106	74.6	1	
Child and parents	126	38.9	198	61.1	1.9	1.2–3.0
**Daily frequency of non-retentive foods (liquid) with sucrose** ^α^						
≤ 7 times/day	66	29.5	158	70.5	1	
> 7 times/day	96	39.7	146	60.3	1.6	1.1–2.4
**dmft index**						
dmft < 5	153	34.2	295	65.8	1	
dmft ≥ 5	9	51.0	9	50.0	1.9	0.7–5.5

Symbol (^*^) indicates the question asked to parents during the interview.

Symbol (^α^) means statistically significant association (p < 0.05).

**Table 3:** Distribution of children using bivariate analysis with oral hygiene as dependent variable

Dependent variable: Oral hygiene						
Variables	Deficient oral hygiene (n = 116)		Regular oral hygiene (n = 350)			
	n	%	n	%	OR	95% CI
**Age** ^α^						
6 years old	47	18.5	207	81.5	1	
4 years old	69	32.5	143	67.5	2.1	1.4–3.3
**Number of siblings** ^α^						
< 3	91	22.9	306	77.1	1	
≥ 3	25	36.2	44	63.8	1.9	1.1–3.4
**Age of children that received help during toothbrushing** ^α^						
> 4 years	63	22.0	224	78.0	1	
≤ 4 years	53	29.6	126	70.4	1.5	1.0–2.4

Symbol (^α^) means statistically significant association (p < 0.05).

**Table 4:** Chi-square (_X_^2^) tendency analysis according to dental caries and *S. mutans* levels for variables considered very relevant in the study

Dependent variable: Dental caries						
Variables	With dental caries (n = 112)		Without dental caries (n = 354)		OR	95% CI
	n	%	n	%		
**Family income (in US$)** ^α^						
≥ 730.40	4	8.9	41	91.1	1.0	1
≥ 365.20–< 730.40	16	20.0	64	80.0	2.6	0.8–8.2
≥ 146.10–< 365.20	44	21.9	157	78.1	2.9	0.1–8.4
< 146.10	48	34.3	92	65.7	5.4	1.8–15.8
_X_^2^ tendency					13.37	p < 0.0002
**Age of children (in months) that received parental help during toothbrushing** ^α^						
> 60	76	28.1	194	71.9	1.0	1
> 48–≤ 60	10	15.6	54	84.4	0.5	0.2–1.0
> 36–≤ 48	25	19.4	104	80.6	0.6	0.3–1.0
≤ 36	1	33.3	2	66.7	1.3	0.1–14.0
X^2^ tendency					4.03	p = 0.04
Dependent variable: *Streptococcus mutans* level in saliva						
Variables	CFU ≥ 10^6^/ml (n = 162)		CFU < 10^6^/ml (n = 304)		OR	95% CI
	n	%	n	%		
**Oral hygiene** ^α^						
Regular, satisfactory	107	32.6	243	69.4	1	
Very bad, deficient	55	47.4	61	52.6	2.1	1.3–3.2
**Oral hygiene** ^α^						
Satisfactory	19	26.0	54	74.0	1.0	1
Regular	88	31.8	189	68.2	1.3	0.7–2.4
Deficient	55	47.4	61	52.6	2.6	1.4–4.8
X^2^ tendency					10.54	p = 0.0012

Symbol (^α^) means statistically significant association (p < 0.05).

### Multivariate Analysis

The final model selected the variables with statistical significance of p < 0.20 (Table 5). Those that had statistically significant positive association with the onset of dental caries were: family income inferior to US$110.00, flossing after 24 months of age, no oral hygiene after bottle-feeding, child’s age at the time of discontinuation of bottle-feeding, parents’ difficulties following the programme’s recommendations, sucrose ingestion by children younger than 3 years old, and the parents’ belief that their child would have dental caries someday. High OR values also were observed, but with an inferior CI 95% limit for children who did not practice regular oral hygiene after bottle-feeding and for those who abandoned bottle-feeding after 4 years old.

**Table 5:** Multivariate analysis for factors associated with: dental caries, high level of *Streptococcus mutans* in saliva and unsatisfactory oral hygiene.

Dependent variable: Dental caries				
Variables	Dental caries			
	Crude OR	95% CI	Adjusted OR	95% CI
**Family income (in US$)**				
≥ 110.00	1		1	
< 110.00	2.1	1.2–3.6	2.4	1.2–4.6
**Age at the start of dental floss use**				
< 24 months old	1		1	
≥ 24 months old	1.8	1.0–3.2	2.5	1.1–5.5
**Child’s oral hygiene after bottle-feeding**				
Yes	1		1	
No, sometimes	1.8	1.0–3.2	2.0	1.0–3.8
**Child’s age when bottle-feeding habit was abandoned**				
≤ 4 years old	1		1	
> 4 years old	1.7	0.9–3.1	2.1	1.0–4.4
**Parents’ difficulties to follow the Preventive Program’s recommendations**				
No	1		1	
Yes	1.8	1.1–3.1	2.6	1.4–5.1
**Parents follow the recommendations on sucrose intake before the child is 3 years old**				
Yes	1		1	
No/sometimes	3.4	1.7–6.7	2.9	1.4–5.9
**Question for the child’s parents: Will your child have dental caries someday?**				
No	1		1	
Yes	1.9	1.2–3.0	2.1	1.2–3.7
Dependent variable: *Streptococcus mutans* level in saliva				
Variables	CFU ≥ 10^6^ ml			
	Crude OD	95% CI	Adjusted OR	95% CI
**dmft index**				
dmft < 5	1		1	
dmft ≥ 5	1.9	0.7–5.5	2.3	0.9–6.2
**The one responsible for the child’s toothbrushing**				
Parents	1		1	
Child/parents	1.9	1.2–3.0	1.8	1.2–2.9
**Oral hygiene**				
Regular, satisfactory	1		1	
Very bad, deficient	2.1	1.3–3.2	2.1	1.4–3.3
**Daily frequency of non-retentive foods (liquid) with sucrose**				
≤ 7	1		1	
> 7	1.6	1.1–2.4	1.6	1.1–2.4
Dependent variable: Oral hygiene				
Variables	Deficient oral hygiene			
	Crude OD	95% CI	Adjusted OD	95% CI
**Child’s age**				
6 years old	1		1	
4 years old	2.1	1.4–3.3	2.2	1.4–3.4
**Number of siblings**				
< 3	1		1	
≥ 3	1.9	1.1–3.4	2.0	1.2–3.5

High SM levels in saliva were positively associated with the dmft index, toothbrushing without parental assistance, deficient oral hygiene and daily ingestion (more than 7 times/day) of non-retentive sweet foods. There was an OR increase for dmft, but the CI (95%) had an inferior 0.9 limit. The factors that showed statistically significant positive association with deficient oral hygiene were: 4 years old and number of siblings equal to or greater than three. Both variables showed an OR increase in relation to the crude value after the adjustment procedure.

## Discussion

In this study, despite PDSC’s support concerning the preventive approach since the first year of life, some children developed dental caries. Thus, it is important to note that of the 112 children with the disease, 84% had satisfactory oral health, showing a positive approuch of program. The disease’s onset could have happened due to the cultural and socioeconomic characteristics associated with deficient oral hygiene and high frequency of sucrose ingestion. Social and behavioural factors are believed to indirectly influence the disease’s onset^26^ and, unfortunately, these factors are difficult to delimit and control because they are tied to family income and educational standards.^25,26^ According to the data analysed, dental caries had a positive relation with the quality of oral hygiene associated with behaviour, socioeconomic and cultural factors, confirming the first hypothesis. The children’s age at the start of dental floss use was a factor that contributed to the presence of caries, seeing as the disease was more often found in children who started the habit after completing 2 years of age. These data could be considered very relevant because the control of dental caries on proximal surfaces in toddlers and adolescents is still a challenge for many oral health programmes, and flossing could aid the maintenance of oral health.^8,19^ The influence of socioeconomic and cultural characteristics was also noted in the inversely proportional association between risk and family income, corroborating other studies.^5,7^ This study also found that a large family with more than three children was a determinant factor for deficient oral hygiene, and it is known that, in poor communities, a high number of siblings is associated with low income, which in turn leads to the onset of dental caries.^12^

In relation to behaviours and diet, prolonged bottle-feeding of children over 4 years old showed association with the onset of dental caries. Furthermore, high ingestion of non-retentive sweet foods showed association with high SM levels in saliva. These findings corroborate the literature that showed the contribution of inappropriate bottle-feeding practices to the onset of dental caries in toddlers,^5^ and reinforced the importance to instruct caregivers about this practice,^9^ especially in relation to the performance of the child’s oral hygiene since the first eruptions of primary teeth.^1^ Another factor observed regarding the family’s behaviours was the association between dental caries and the caregiver’s difficulties following the preventive programme’s recommendations. The difficulties to control the sucrose ingestion of children younger than three were more significantly associated with the onset of dental caries. In this study, the high frequency of ingestion of non-retentive sweet food (more than 7 times/day) showed positive association with the onset of dental caries, corroborating the literature.^6^ In addition, children who are 4 and 5 years old have more independence, which could in turn influence their preference for sweet foods^28^; moreover, the children’s sugar ingestion may be underestimated by their parents, because sucrose could be present in other food sources, contributing to diseases such as obesity and dental caries.^25^ Regarding the parent’s conception of whether their child could develop dental caries someday, the parents who confirmed this had children with caries. This belief suggested some lack of encouragement to adopt the oral care practices recommended by the preventive programme, and the assumption that dental caries would be an inevitable disease. Thus, it is also suggested that oral preventive programmes apply simplified methods with easy understanding, encouraging the target population to seek the best conditions for the promotion of oral health.

In relation to high SM levels and dental caries, positive associations were found in this study for dmft index, sucrose ingestion frequency, lack of the parent’s support in the child’s oral hygiene and quality of oral hygiene, confirming the second hypothesis. This study showed a proportional increase between SM levels with biofilm index and dmft superior to five, corroborating the literature concerning the closer relation between the high onset of dental caries and the presence of specific microorganisms.^3,22^ Low SM levels were found in children whose toothbrushing was performed by the caregivers only, showing the need to improve this practice and encourage the parents to monitor their children at all times during oral hygiene. Given the above, the importance of the caregiver’s supervision during the child’s oral hygiene is emphasised,^13^ especially for children aged 4 years old, because irregular biofilm removal associated with a cariogenic diet could contribute to the increase in the onset of caries.^1^ In this way, the presence of biofilm, as indicator of the risk of caries, could be an important tool for education in oral health.^17,18,26^ Therefore, improving toothbrushing techniques at the home environment with inclusion of flossing since the first teeth’s eruption may be considered an essential item for the prevention of dental caries.

In summary, this study showed a positive association between the oral hygiene index, SM levels, ingestion of sweet foods and the onset of dental caries, corroborating the modern conception of dental caries as a biofilm and sucrose-dependent disease.^26^ Behaviour and socioeconomic factors such as family income, parent’s absence of compliance in relation to the child’s oral health, age at the time of abandonment of the bottle-feeding habit, frequency of ingestion of sweet foods before the child is 3 years old and late start of flossing were indirectly associated with the onset of caries. The results obtained with the plaque index and SM levels could encourage the implementation of low-cost oral health programmes since the first year of life. The simplified oral hygiene index used is associated with the SM levels in saliva and seems to be an efficient method to evaluate children’s oral health, being adopted as a strategy for control of biofilm and reduction of cariogenic microorganism in the saliva of preschoolers. The biofilm evidenced could help dentists instruct patients in relation to brushing and flossing techniques, which are considered important tools for education in oral health. Despite the indirect influence of socioeconomic and cultural factors on the onset of dental caries, these factors require special attention due to the difficulties to control them, and educative actions would be necessary to promote changes in the community’s behaviour.

The knowledge about factors associated with the onset of dental caries obtained in this study could support the structuring of a clinical protocol for evaluation of the risk of caries, particularly useful in children. The determination of this risk is important to implement prevention actions before the disease manifests itself. Thus, the decisions related to treatment based on the risk of caries and on the parents’ compliance are essential elements of the contemporary clinical care of infants, children and adolescents.

## Conclusion

High SM levels in saliva, deficient oral hygiene and high frequency of sucrose ingestion were associated with dental caries in children. In addition, cultural, socioeconomic and behavioural factors indirectly influenced the onset of dental caries.
